# Similarities and Differences Between COVID-19-Related Multisystem Inflammatory Syndrome in Children and Kawasaki Disease

**DOI:** 10.3389/fped.2021.640118

**Published:** 2021-06-18

**Authors:** Min-Sheng Lee, Yi-Ching Liu, Ching-Chung Tsai, Jong-Hau Hsu, Jiunn-Ren Wu

**Affiliations:** ^1^Division of Pediatric Infectious Disease, Department of Pediatrics, Kaohsiung Medical University Hospital, Kaohsiung, Taiwan; ^2^Division of Pediatric Cardio-Pulmonology, Department of Pediatrics, Kaohsiung Medical University Hospital, Kaohsiung, Taiwan; ^3^Department of Pediatrics, E-Da Hospital, Kaohsiung, Taiwan; ^4^School of Medicine, I-Shou University, Kaohsiung, Taiwan; ^5^Department of Pediatrics, School of Medicine, College of Medicine, Kaohsiung Medical University, Kaohsiung, Taiwan

**Keywords:** coronavirus disease 2019, multisystem inflammatory syndrome in children, pediatric multisystem inflammatory syndrome temporally associated with SARS-CoV-2, Kawasaki disease, difference

## Abstract

In December 2019, the first case of coronavirus disease (COVID-19) was first reported in Wuhan, China. As of March 2021, there were more than 120 million confirmed cases of COVID-19 and 2.7 million deaths. The COVID-19 mortality rate in adults is around 1–5%, and only a small proportion of children requires hospitalization and intensive care. Recently, an increasing number of COVID-19 cases in children have been associated with a new multisystem inflammatory syndrome. Its clinical features and laboratory characteristics are similar to those of Kawasaki disease (KD), KD shock syndrome, and toxic shock syndrome. However, this new disorder has some distinct clinical features and laboratory characteristics. This condition, also known as multisystem inflammatory syndrome in children (MIS-C) associated with COVID-19, has been observed mostly in Europe and the United States. This emerging phenomenon has raised the question of whether this disorder is KD triggered by SARS-CoV-2 or a syndrome characterized by multisystem inflammation that mimics KD. This narrative review is to discuss the differences between MIS-C and KD with the aim of increasing pediatricians' awareness of this new condition and guide them in the process of differential diagnosis.

## Introduction

In December 2019, the first case of coronavirus disease (COVID-19), caused by the novel human coronavirus severe acute respiratory syndrome coronavirus 2 (SARS-CoV-2), was first reported in Wuhan, Hubei province, China (1). It rapidly spread to more than 215 countries, including those in Asia, Europe, North America, and South America and has become an emergent health crisis globally. The outbreak of COVID-19 worldwide had been declared as a pandemic. As of March 28, 2021, there were more than 120 million confirmed cases of COVID-19 and 2.7 million deaths. The clinical manifestations of COVID-19 range from asymptomatic to severe pneumonia, respiratory failure, acute respiratory distress syndrome, and fatal outcomes. A systemic review of all COVID-19 literatures published between January 1, 2020 and March 18, 2020, demonstrated that only 1–5% COVID-19 cases have been reported in children (2). The prevalence of laboratory-confirmed COVID-19 cases in children younger than 15 years reported to The European Surveillance System is only 2.1% (3). Compared with adults with COVID-19, children with COVID-19 present milder symptoms and less mortality. Among the 171 laboratory-confirmed COVID-19 pediatric patients treated at Wuhan Children's Hospital, only 1.7% pediatric patients received intensive care support or mechanical ventilation (4). In a large scale study of pediatric COVID-19, only 5.9% of them had hypoxemia, compared with adults with COVID-19 (18.5%), and 0.6% had a life-threatening condition (5).

Recently, a new syndrome characterized by a systemic inflammatory status that seems to be related with SARS-CoV-2 infection has been identified. This syndrome, also known as multisystem inflammatory syndrome in children (MIS-C), seems to significantly overlap with Kawasaki disease (6–8). Although several common symptoms are noticed in both KD and MIS-C, including skin rash, lymphadenopathy, strawberry tongue, and an elevation of inflammatory biomarkers, MIS-C demonstrates unique features. Belhadjer et al. reported the tendency of older onset, prominent gastrointestinal symptoms, and more left ventricular systolic dysfunction (6). Therefore, this narrative review is to discuss differences between MIS-C and KD, and to alert pediatricians about this emergent and aggressive disease, MIS-C, in children with COVID-19.

## SARS-CoV-2 Related Multisystem Inflammatory Syndrome in Children

In April 2020, general practitioners in London received a warning from the National Health Service in England to be cautious of increasing numbers of children suffering from a multisystem inflammatory state with overlapping presentations of toxic shock syndrome and atypical KD (9). Despite no direct confirmation of SARS-CoV-2 or exposure history, children with COVID-19 developed fever, hypotension, severe abdominal pain, and cardiac dysfunction (10). Attention should be given to features of the cytokine storm, particularly high serum interleukin-6 levels, and patients generally require inotropic support to increase cardiac output with a rare need for extracorporeal membrane oxygenation (10). In addition, in Bergamo, Italy, KD was diagnosed in 19 children over a short period, roughly equivalent to the total number of cases previously diagnosed over 3 years in that region (8). Similarly, the French Health Minister reported that 16 children with symptoms of KD were hospitalized in Paris hospitals (11). A series of cases involving children with this condition have been reported in the UK (9), Italy (8), Spain (12), France, Switzerland (6), and the United States (13). The Center for Disease Control and Prevention of the United States has developed a case definition and has specifically named the condition MIS-C. To date, three definitions of MIS-C have been proposed, by Center for Disease Control and Prevention of the United States, the Royal College of Pediatrics and Child Health, and the World Health Organization, respectively (14–16). Although there are some discordances between these definitions (Table 1), the common characteristics are fever, evidence of inflammation, multisystem organ involvement, likely contact or evidence of SARS-CoV-2 infection, and exclusion of other microbial causes. Indeed, these definitions may overlap partial criteria for KD (17).

**Table 1:** Case definitions of multisystem inflammatory syndrome in children (14–16).

	**CDC (United States)**	**Royal College of Pediatrics and Child Health (United Kingdom)**	**WHO**
Name	Multisystem inflammatory system in children (MIS-C) associated with COVID-19	Pediatric inflammatory multisystem syndrome temporally associated with SARS-CoV-2 (PIMS-TS)	Multisystem inflammatory syndrome in children and adolescents with COVID-19
Age	<21 years	Child	0–19 years
Fever	Fever >38°C (or Report of subjective fever) for ≥24 h	Persistent fever >38.5°C	Fever ≥3 days
Clinical features	Severe illness requiring hospitalization, with Multisystem involvement (≥2 organ systems involved, cardiovascular, respiratory, renal, neurologic, hematologic, gastrointestinal, dermatologic)	Single or multi-organ dysfunction (shock, cardiac, respiratory, renal, gastrointestinal or neurologic disorder) with additional features	At least two of the following: (1) Rash, bilateral non-purulent conjunctivitis, or muco-cutaneous inflammation signs. (2) Hypotension or shock. (3) Cardiac dysfunction, pericarditis, valvulitis, or coronary abnormalities. (4) Coagulopathy. (5) Acute gastrointestinal symptoms.
COVID-19 infection	Positive SARS-CoV-2 RT-PCR, or positive serology, or positive antigen test, or COVID-19 exposure within the 4 weeks before onset of symptoms	SARS-CoV-2 PCR positive or negative	Positive SARS-CoV-2 RT-PCR, or positive serology, or positive antigen test, or COVID-19 exposure
Exclusion	No alternative plausible diagnosis	Any other microbial cause	No other obvious microbial cause of inflammation

*CDC, Centers for Disease Control and Prevention; COVID-19, Coronavirus disease; SARS-CoV-2, Severe acute respiratory syndrome coronavirus 2; RT-PCR, Reverse transcription polymerase chain reaction; WHO, World Health Organization*.

The geographical distribution of MIS-C cases overlaps with that of COVID-19 cases. Most MIS-C pediatric cases in New York and United Kingdom did not have direct evidence of acute COVID-19 infection (18–20). However, most of them had positive serological results, especially IgG, suggesting that MIS-C could represent a delayed immune response to SARS-CoV-2. The hypothesis that MIS-C is a post-infectious manifestation was further supported by a lag time of 4–5 weeks in the epidemic curve of COVID-19, followed by that of MIS-C (18, 21). Therefore, countries with currently ongoing COVID-19 epidemic should consider this rare but critically delayed syndrome in children.

## Differences Between MIS-C and KD

Some clinical similarities between MIS-C and KD have been found. The most noteworthy common feature is febrile illness involving inflammation of the blood vessels and possible subsequent consequences of coronary artery aneurysms. In addition, patients with MIS-C may have some similar features of KD including fever, conjunctivitis, rash, and congestion of the oropharynx. However, these non-specific clinical findings could be observed in many pediatric infectious diseases. Therefore, it is unclear whether or to what extent MIS-C and KD may overlap. Indeed, these two syndromes appear to have some distinct differences, including age distribution, racial and ethnic disparities, clinical manifestations, cardiac involvement, inflammatory markers, treatment and outcome (Table 2) (18, 19, 22–25).

**Table 2:** Difference between multisystem inflammatory syndrome in children and Kawasaki disease (18, 19, 22–25).

	**MIS-C**	**KD**
Age	Older children and adolescent, Median age 8–11 years	Infant and young children, 76% of affected children <5 years
Sex ratio	Male/Female 1:1 to 1.2:1	Male/Female 1.5:1 to 1.7:1
Race and ethnicity	Black and Hispanic descent	Asian descent
Gastrointestinal symptoms	Very Common (53–92%)	Less common (≈20%)
Myocardial dysfunction and shock	Common, 73% elevated BNP, 50% elevated troponin levels, 48% receive vasoactive support	Less common, 5% receive vasoactive support
Organ dysfunction	Multiorgan dysfunction common	Multiorgan dysfunction not common
Inflammatory markers	Highly elevated CRP, ferritin, procalcitonin, and D-dimer, lymphopenia and thrombocytopenia	Elevated CRP, D-dimer, and thrombocytosis, usually normal ferritin; thrombocytopenia is rare
Treatment	IVIG, Corticosteroids, IL-1 blocker, IL-6 inhibitors	IVIG, Corticosteroids, IL-1 blockers
Outcome	Fatality rate 1.4–1.7%	Fatality rate 0.01%

*MIS-C, multisystem inflammatory syndrome in children; KD, Kawasaki disease; BNP, B-type natriuretic peptide; CRP, C-reactive protein; IVIG, intravenous immunoglobulin*.

### Incidence and Epidemiology

The incidence of KD is markedly higher in East Asia, especially in Japan, Korea and Taiwan, but low in Europe and the United States. The annual incidence among children younger than 5 years old was ~240 per 100,000 in Japan and 20 per 100,000 in United States, respectively (24). While the incidence of MIS-C is uncertain, it was mostly reported in Europe (18, 26, 27) and the United States (20, 26). Intriguingly, much fewer cases had been reported in Asian countries, including Iran (28), Korea (29), and India (30). Regardless, MIS-C appears to be a relatively rare complication of COVID-19 in children. According to a study in France, the risk of MIS-C was fewer than 0.2 cases per 100,000 children (3). In addition, another report from United States shows the incidence of MIS-C about 2 per 100,000 children (18).

The epidemiology of KD has been similar worldwide for the past decades, with 50% cases occurring in children younger than 2 years, 80% case younger than 5 years, with a peak incidence at 6–11 months of age (31). There was a significantly higher incidence of KD in boys than in girls. Male patients represented 56–70% of KD patients (24, 32). This contrasts with the epidemiology of MIS-C, which usually affects older children and adolescents. In fact, age distribution in a pediatric study involving 156 MIS-C cases showed a median of 8 years and an interquartile range of 5–11 years (3). The balanced or mild male dominant sex ratio in MIS-C is also different from those observed in KD. A systemic review of 16 case series of total 655 MIS-C cases reported the male to female ratio 1.2:1 (33) and another study of total 270 MIS-C cases reported close to 1:1 (34).

KD apparently shows a predilection in Asian ancestry, even with transmigration (35). A retrospective analysis of KD children in United States demonstrated annual incidence was highest among Asians and Pacific Islander (30 per 100,000), intermediate among non-Hispanic African Americans (17 per 100,000) and Hispanic (16 per 100,000), and lowest among Caucasians (12 per 100,000) (36). In contrast to KD, children of African or Hispanic descent carried higher risk of MIS-C while Asian children accounted for less cases (9). In parallel, two large studies of MIS-C in the US demonstrated that 25–40 percent of patients were black, 31–36% Hispanic and 5% Asian (19, 37). To date, no cases of MIS-C have been reported in China and Japan (38). Thus, the underlying genetic discrepancy might be a factor resulting in these epidemiologic differences.

### Physiopathology

KD is an acute self-limited systemic vasculitis. The physiopathology is unclear but is considered to be associated with exposure of a genetically pre-disposed child to an unknown infectious agent (24). This exposure may trigger abnormal immune reactions, leading to increased Th1/Th17-related immunity and a Treg/Th17 imbalance, which ultimately results in the release of large amounts of proinflammatory cytokines and systemic inflammation in all the medium-sized arteries and in multiple organs and tissues (35).

The physiopathology of MIS-C is also not well-understood, but it appears to be a consequence of massive release of inflammatory mediators with exaggerated activation of the immune system like cytokine storm (39). Based on the timing of the increased MIS-C cases relative to the peak of COVID-19 pandemic, post-infectious process is suggested (18, 21). Recently, small bowel mucosal biopsy from a COVID-19 patient with vasculitis demonstrated the presence of SARS-CoV-2 in endothelial cells (40). This finding corroborates that SARS-CoV-2 infection might be a pre-requisite for the development of MIS-C. In addition, detection of autoantibodies in MIS-C patient in mucosal and cardiac tissue, endothelial cells and cytokine molecules have been reported recently (41, 42), similar to patients with KD (43). Autoantibodies engaging with Fcγ receptors on neutrophils and monocytes resulting in formation of immune complexes are probably contributing to disease pathogenesis in both MIS-C and KD (41, 42). However, the underlying mechanisms for hyperinflammation are different between KD and MIS-C. In KD, interleukin-1 (IL-1) has direct inflammatory effects on coronary endothelial cells (35), while in MIS-C, the myocardial dysfunction and higher severity of the SARS-CoV-2 infection is predominantly driven by IL-6 and IL-10 (44).

### Clinical Characteristics

Fever is a universal feature in KD patients. Manifestations of oral mucositis, conjunctivitis, extremity change and polymorphous rash are seen in ~90% of cases, while cervical lymphadenopathy in ~30% and diarrhea only in ~20% (45). Shock, ventricular dysfunction and ischemic damage are rarely reported in the acute stage of KD (46). Only <3% of KD patients developed shock (47).

Compared with KD, the clinical features of MIS-C include a higher incidence of gastrointestinal symptoms, multisystem organ involvement, and relatively fewer classical KD symptoms (44). In large case series (18–20, 22, 37), most patients with MIS-C have similar clinical symptoms including fever (100%), gastrointestinal symptoms like abdominal pain, vomiting and diarrhea (53–92%), skin rashes (45–60%), conjunctivitis (29–56%), mucosal change (29–35%) and respiratory involvement (21–63%). A study of 186 MIS-C patients in the US, 71% patients have involvement of at least four organ systems (19). The most common involved organ systems are the gastrointestinal (92%), cardiovascular (80%), hematologic (76%), mucocutaneous (74%), and respiratory systems (70%). Intriguingly, most MIS-C patients present with 5 or more days of fever (78%) and about 40% of patients meet the diagnostic criteria of KD or incomplete KD (19). However, 35–50% MIS-C patients experience hypotension (18, 20), and neurologic symptoms are common. In a report of 616 patients with MIS-C, neurologic involvement was found in 20% (48). Indeed, life-threatening neurologic conditions including severe encephalopathy, stroke, Guillain-Barré syndrome, and acute fulminant cerebral edema were observed in 20 patients (3.2%).

In general, COVID-19 is a self-limited disease in children, and most studies on clinical characteristics of COVID-19 in children do not report features consistent with KD (5, 49, 50). For example, Parri et al. found that fever (>38°C) was present in only 41% of cases, while rash was present in only 3% of cases (49). Therefore, only those COVID-19 children who has prolonged fever and other features of KD should be considered as MIS-C.

### Blood Markers

Systemic inflammation is characteristic laboratory finding of KD. Typical manifestations included elevation of C-reactive protein, erythrocyte sedimentation rate, leukocytosis, increased immature neutrophil and thrombocytosis. High ferritin, thrombocytopenia and high triglycerides are seen in macrophage activation syndrome, a rare complication of KD (23).

In MIS-C patients, increased inflammatory markers including C-reactive protein, procalcitonin, ferritin, and serum IL-6, are also widely observed (7, 11, 18, 19, 51, 52). High levels of erythrocyte sedimentation rate and thrombocytopenia were also common (33, 53). Compared to KD, patients with MIS-C usually have lower platelet and lymphocytes, higher ferritin and procalcitonin level (11, 18, 51, 54). MIS-C symptoms have a greater resemblance to those of macrophage activation syndrome, presenting with elevated ferritin, D-dimer, triglyceride levels, with the cytokine storm (44, 52). However, the cytokines underlying the inflammatory mechanisms appear to be distinct in MIS-C and KD. In KD, IL-1 appears to be the main mediator of coronary artery inflammation, while IL-6 and IL-8 are main mediators in MIS-C (55).

### Cardiac Manifestations

In KD, the cardiac hallmark is coronary artery abnormalities (44). Coronary artery aneurysms occur in 20–25% of untreated KD children (24). Evidence of mild to moderate ventricular dysfunction, valvular regurgitation and pericardial effusion are commonly seen during the acute stage of KD. Treatment with intravenous immunoglobulin (IVIG) usually restores normal ventricular function rapidly. Shock and severe myocardial dysfunction are rare in KD (46). One study found that only 138 of 9,488 children with KD were complicated by shock (56). Increased risk of heart complications is found particularly in these KD patients with shock. For example, left ventricular systolic dysfunction was detected only in 4% of patients with KD without shock, but in 31% of KD patients with shock (57).

By contrast, cardiac complications of MIS-C are common, including myocardial dysfunction, manifested by imaging and higher B-type natriuretic peptide (BNP) and troponin levels. In fact, left ventricular dysfunction was reported in 35–100% of children with MIS-C (58). Belhadjer et al. reported that 80% of patients with MIS-C who developed acute left ventricular failure required inotropic support, 28% received extracorporeal membrane oxygenation support (6). Unlike classic KD, coronary arteries involvement was less common in MIS-C. In a case series of 503 patients with MIS-C, only 13% of them had coronary artery aneurysms (CAA) (59), and most of these CAA (93%) were mild and there were no large or giant aneurysm.

### Diagnostic Criteria

With the overlapping clinical manifestations and the lack of a specific diagnostic test for either MIS-C or KD, distinguishing the two conditions in an individual patient can be challenging. Diagnosis of KD according to the criteria established by Dr. Tomisaku Kawasaki in 1967 is based on the presence of ≥5 days of fever and the presence of ≥4 of the 5 principal clinical features, bulbar conjunctivitis, oral mucous membrane changes, peripheral extremity changes, polymorphous skin rash and cervical lymphadenopathy (24). The criteria used for MIS-C case definition vary between different health agencies (Table 1). Only 25% MIS-C patients' cardiovascular manifestations fulfill criteria for KD (60) and 66% for incomplete KD (61). Thus, MIS-C appears to be a larger group of heterogeneous diseases, including SARS-CoV-2 induced cytokine storm syndrome (CSS), COVID-19 with severe inflammatory responses, and KD concurrent with SARS-CoV-2 infection (20).

### Treatment

The recommended initial therapy of KD includes IVIG (2 g/kg) administered as a single infusion and oral aspirin. The use of corticosteroids has been controversial for children with KD. Systemic corticosteroid is additionally added in patients who are at high risk for IVIG resistance (24). Second dose of IVIG, high-dose pulse steroids or monoclonal antibody against TNF-α (infliximab) is considered for IVIG-resistant patients. For treatment of highly refractory KD, human IL-1 receptor antagonist (anakinra), cytotoxic agents, or rarely plasma exchange may be considered (24).

On the other hand, since without widely accepted guidelines, treatment strategies for MIS-C are similar with the standard therapy of KD, including IVIG and corticosteroids (33, 53). For refractory patients of MIS-C, monoclonal antibodies to the IL-6 receptor (tocilizumab), IL-1 receptor antagonist (anakinra), monoclonal antibody against TNF-α (infliximab) or convalescent plasma therapy have been used. In a retrospective study of 111 MIS-C patients, 65% received initial treatment with IVIG alone and 31% received IVIG plus methylprednisolone (62).

### Prognosis

The prognosis of KD is better than MIS-C. The mortality rate of MIS-C is 1.7% in the US and 1.4% in Europe (33), which is much higher than 0.01% reported in children with KD in Japan (63). Timely treatment for KD patient reduces the CAAs from 20–25 to 5% or less (24). Overall, 50% of CAAs regress to normal by 1–2 years and CAA regression occurred in 75% of patients by 30 years (64). The long-term prognosis for coronary arterial aneurysm in MIS-C remains unknown but almost all patients with increased cardiac enzymes and left ventricular dysfunction had full recovery (13, 55).

## Conclusions

There are both similarities and differences in the epidemiology, manifestations, treatment, and prognosis between MIS-C and KD. The genetic susceptibility resulting in KD and MIS-C remain to be determined, but it will be worthwhile to evaluate in the future. Give with the standard therapies for KD, delays or failures in the diagnosis of KD may lead to unfavorable outcomes related to coronary aneurysms. Thus, MIS-C patients should be closely observed, and correctly distinguishing these two diseases is of the utmost importance to the pediatricians caring children with COVID-19.

## Author Contributions

M-SL wrote the first version, complemented by the work of Y-CL and J-HH. J-HH, C-CT, and J-RW revised and performed modifications and lapidating the final work. All authors made substantial contributions to the project, participating in all phases.

## Conflict of Interest

The authors declare that the research was conducted in the absence of any commercial or financial relationships that could be construed as a potential conflict of interest.
